# ViTDroid: Vision Transformers for Efficient, Explainable Attention to Malicious Behavior in Android Binaries

**DOI:** 10.3390/s24206690

**Published:** 2024-10-17

**Authors:** Toqeer Ali Syed, Mohammad Nauman, Sohail Khan, Salman Jan, Megat F. Zuhairi

**Affiliations:** 1 Faculty of Computer and Information Systems, Islamic University of Madinah, Madinah 42351, Saudi Arabia; toqeer@iu.edu.sa; 2 Department of Computer Science, Effat College of Engineering, Effat University, Jeddah 22332, Saudi Arabia; mnauman@effatuniversity.edu.sa (M.N.); sohkhan@effatuniversity.edu.sa (S.K.); 3 Department of Information Technology, Alburaimi University College, Alburaimi 512, Oman; salman@buc.edu.om; 4 College of Computer Studies, University of Technology Bahrain, Salmabad 18041, Bahrain; 5 Malaysian Institute of Information Technology, Universiti Kuala Lumpur, Kuala Lumpur 50250, Malaysia

**Keywords:** malware, vision transformers, android, security

## Abstract

Smartphones are intricately connected to the modern society. The two widely used mobile phone operating systems, iOS and Android, profoundly affect the lives of millions of people. Android presently holds a market share of close to 71% among these two. As a result, if personal information is not securely protected, it is at tremendous risk. On the other hand, mobile malware has seen a year-on-year increase of more than 42% globally in 2022 mid-year. Any group of human professionals would have a very tough time detecting and removing all of this malware. For this reason, deep learning in particular has been used recently to overcome this problem. Deep learning models, however, were primarily created for picture analysis. Despite the fact that these models have shown promising findings in the field of vision, it has been challenging to fully comprehend what the characteristics recovered by deep learning models are in the area of malware. Furthermore, the actual potential of deep learning for malware analysis has not yet been fully realized due to the translation invariance trait of well-known models based on CNN. In this paper, we present ViTDroid, a novel model based on vision transformers for the deep learning-based analysis of opcode sequences of Android malware samples from large real-world datasets. We have been able to achieve a false positive rate of 0.0019 as compared to the previous best of 0.0021. However, this incremental improvement is not the major contribution of our work. Our model aims to make explainable predictions, i.e., it not only performs the classification of malware with high accuracy, but it also provides insights into the reasons for this classification. The model is able to pinpoint the malicious behavior-causing instructions in the malware samples. This means that our model can actually aid in the field of malware analysis itself by providing insights to human experts, thus leading to further improvements in this field.

## 1. Introduction

Android has emerged as one of the most popular software stacks for touch screen devices, such as smartphones and tablets, in just a little over a decade. It has constantly maintained top market share over the past decade. Over 71% of all smartphones sold to consumers in the second quarter of 2022 were running the Android operating system, according to a report by Statista [1].

The open source development and distribution is one of the key distinctions between the Android software stack and those of its rivals. The entire Android software stack, which consists of an operating system, middleware, and some integrated applications, is kept open source. Many developers and contributors are striving to improve the architecture of Android thanks to the platform’s open source nature. The first open source mobile phone software stack that can be purchased and sold off-the-shelf is Android, which, in contrast to earlier open source mobile phone strategies, has focused on the consumer market. The flip side of this positive aspect is that Android’s open nature also draws many malicious programs that try to steal users’ personal information or perform otherwise malicious activities on these devices [2]. For instance, these devices include a myriad of sensors that make it simple to track users’ whereabouts and physical activities [3].

We notice that there have always been time constraints for security experts when it comes to malware detection. Experts are always trying to catch up with the newest 0-days and malicious entities figure out a way of avoiding the detection algorithms. One of the more recent developments in AI is giving them a leg-up when it comes to identifying malware. Learning from samples has been a big step in battling malware on Android. Machine learning has had great success in mitigating this issue, and deep learning models are succeeding as well. However, almost all of the existing malware detection techniques to date utilize some variation of the convolutional operations as the core engine for ML models [4].

While effective against many simplistic known and 0-day malware families, CNNs have a significant limitation: they detect individual features but the temporal and spatial relations between these features are lost [5]. To explain this limitation in the domain of computer vision for instance, consider if a picture containing a tyre, hood, bumpers and windows is fed to a car-detecting CNN. The model will probably categorize it as a car. The reason is that while individual features are detected by the CNN, there is no mechanism of establishing the interconnectedness of the features.

The second issue with CNNs is the lack of establishing causation [6]. For instance, if a picture of a race track with several cars is fed to the CNN model described above, it will correctly recognize it as having a car but no information can be gathered from the model regarding why this classification was made. The model will not be able to identify the pixels which led to the conclusion. CNNs’ difficulty in capturing long-range dependencies and contextual relationships lies in Android binaries which lack the ability to understand disjointed but related malicious behavior. This results in challenges in CNNs when dealing with obfuscation, as malware often has non-contiguous patterns that CNNs struggle to identify. In malware detection, translation invariance in CNNs results in the loss of critical contextual relationships between features. For example, when CNNs analyze Android binaries, they identify individual opcode sequences but often fail to connect related instructions that are spatially separated in the binary. This issue arises because CNNs apply convolution filters uniformly across the input, assuming that the same feature is equally important regardless of its position.

However, in the context of malware detection, the order and position of certain opcodes can indicate malicious behavior. For instance, a malicious sequence may start with a benign-looking function but contain a hidden exploit several lines later. CNNs may recognize each part but fail to correlate them, as the model lacks a mechanism to understand dependencies between distant features.

In contrast, vision transformers solve this problem through their attention mechanism, which focuses on the relationships between different parts of the sequence, irrespective of their position. This feature is crucial in identifying malware that employs obfuscation or spreads its malicious activity across various parts of the binary.

In this paper, we note that this establishment of causation is not only important in its own right. We hypothesize that in addition to making the model more explainable—a goal in itself—it can also lead to better classification. One method of establishing such causation is through the use of attention-based models. We note that traditional attention-based models are slow to work on image-based inputs and are simply infeasible for complex, long-form data such as sequences of instructions in malware datasets. Therefore, we present a novel attention-based model centered around the concept of vision transformers [7] for the explainable classification of malware on Android.

Our model works by creating a 2D (10-channel) representation of 1D sequences of opcodes extracted from malicious and benign samples of Android applications. We show that it is possible to use the concepts of attention provided by vision transformers to pinpoint exactly what parts of a sample contribute to the identification as malware. We also show that it is possible to extract previously hidden behavior through the use of 1D-to-2D mapping. Our model not only improves the false positive rate of detection, it also makes the predictions explainable, leading to improvements in the underlying security model itself.

Contributions: The major contributions of this paper can be summarized as follows:

1. We present a novel framework by adapting the vision transformer framework for malware analysis, leading to a model that produces explainable results.
2. We provide details about modifying the underlying vision transformer model itself through several modifications that make training these models more feasible—solving a major issue when working with such large-scale models.
3. We provide an analysis of the explainability aspect of our model and discuss how security experts can use the outputs of the model to improve the domain of malware analysis itself.

Paper Organization: The rest of this manuscript provides details about the domain and background information in Section 2 with specific attention paid to vision transformers in Section 2.3. The details of our model along with several hyperparameters are covered in Section 3. Experiments with large-scale real world malware datasets and their results in terms of model performance and explainability are presented in Section 4. Finally, the paper is concluded and a discussion about the highly promising future directions in this line of research are presented in Section 5.

## 2. Background

To fully understand this research, the reader must be familiar with two domains: Android malware analysis and machine learning—specifically transformers. In this section, we first provide a brief overview of malware analysis for the Android platform and then switch to a discussion of machine learning.

### 2.1. Malware Detection on the Android Platform

Traditional malware analysis requires a human to analyze any given sample either through static analysis or through dynamic sandboxing. The former is easier and can be performed in a fraction of the time required to run a dynamic sandbox and execute the sample for an arbitrarily long amount of time. Static exploration is therefore the most commonly used method for malware analysis [8,9]. This manuscript focuses on this type of analysis as well. The baseline for static analysis was provided by the seminal work of STIDE [10], which discussed exploring the spatiotemporal relationships between different low-level instructions in any executable’s code. This work has been extended for the Android platform by several studies. One of the most prominent one was taint analysis, which aimed to detect information leakage through a deep static analysis of code [11]. This approach was very promising in terms of its technical strengths but required so much security expertise that the average (or even some expert) programmers simply could not use it. Other similar works such as FlowDroid [12] aimed to simplify this but did not manage to achieve the same success rate as TaintDroid. This line of research was quickly abandoned due to infeasibility in terms of deployment. Other mechanisms such as risk–score analysis were proposed by Pen et al. [13]. While this reduced the load on security experts, it was too computationally intensive to be feasible for the large scale. Obfuscation detection and malware reconstruction were proposed by Garcia et al. [14], but these, too, were infeasible due to the number of person-hours required from security experts to study individual malware samples.

As can be seen, the major issue with all the techniques based on expert analysis is that it is a lost cause from the very start. Especially in the case of mobile phone platforms, the incentive is so great for malware writers that it is impossible for any set of human experts to keep pace with the newly created malware surfacing every day. Analysts have therefore turned to machine learning (and specifically deep learning) in the past few years to try and keep up with the ever increasing number of malware. Several studies have been carried out in the past to apply deep learning to the issue of malware analysis in general and for Android in particular. For instance, convolutional neural networks (CNNs) McLaughlin et al. [15] utilized to categorize malware using static analysis of byte-code from a disassembled program. Malware signatures do not need to be explicitly created because the network automatically learns malware suggestive features from raw byte-code sequences [16].

Similar to this, Karbab et al. [17] introduced MalDozer, a framework for Android malware detection based on sequence mining using CNNs. MalDozer creates sequences of vectors using API calls that are retrieved from Android app packages as input. An Android malware detection framework that automatically extracts the Linux kernel system calls for each application and builds weighted directed graphs has been suggested by Hou et al. [18]. They also use a convolutional neural network framework to categorize recently discovered malware. Dynamic analysis methods were proposed by Ali-Gombe et al. [19], Canfora et al. [20] and more recently, by Amin et al. [21] by recognizing the behavioral signature of active applications. Although these and other dynamic analysis techniques [22,23,24] are more obfuscation-resistant, they offer less scalability since they require more processing resources. Backes et al. [25] have utilized the concept of Baysian machine learning to study the issue from a Bayesian perspective, thus baking in the concept of uncertainty to the model. This work is different from others but lacks the computational performance required to scale up to a usable level. Nauman et al. [26] have utilized several models from the deep learning domain, including Boltzman machines and autoencoders, for performing malware analysis. To fully grasp the distinctions between these various authors’ contributions, it is essential to have a foundational understanding of the deep learning models that underpin them. In the following section, we offer a concise overview of deep learning to ensure the reader has the necessary context. Readers already familiar with deep learning concepts may proceed directly to Section 2.2.3.

### 2.2. Brief Aside into Deep Learning

Applications created by malware developers are written in such a way that malware alters its appearance to escape detection. Algorithms are finally employed for labeling to leverage handcrafted features. However, as more malware is created every day, it cannot be efficiently and widely recognized using handcrafted features. End-to-end learning, which refers to machine learning that can automate every step starting from the input to the architecture, extracting features from the input and then performing different operations for its representation to classify the input appropriately, is what deep learning satiates. Deep learning designs include neural networks with layers and dense connections between the nodes. The idea of deep learning has been shown to be extremely effective in several fields [27,28,29]. The following details about most popular deep learning models are offered for the sake of clarity.

A dataset is often divided into a training set and a test set in machine learning. To create a new model that takes samples from the test set as input, runs some calculations based on theta values, and forecasts the ground truth, a process is trained on the training set. Model parameters, commonly referred to as theta values, are changed to improve the model and minimize the energy function. To determine how far the expected values for specific data items are from the actual values, a regression model is fitted. When errors are prevalent, the gradient decline method is used to reduce them.

#### 2.2.1. Learning Through Activation Functions

Similarly, each neuron in the hidden and output layers uses activation functions to determine the importance of the information coming from the preceding layer. There are many different types of activation functions, including linear and sigmoid functions. The following general function is provided by the linear activation function, which introduces linearity into the network: F(x)=ax+b. The most well-known activation function, sigmoid, produces values between 0 and 1. However, because it generates small values, which diverges the learning process as well, it suffers from the vanishing gradient problem [30]. The hyperbolic tangent, tanh, function is another activation function that generates output between −1 and 1.

Tanh functions also experience the vanishing gradient problem at some point during neural training. The ReLu activation function [31] and its various variations are a broad approach frequently used to address this issue. However, ReLU has a major issue that it is not fully differentiable at the origin. This leads to issues during training and contributes to the vanishing gradient problem. An alternative to this is the smoother, fully differentiable alternative called GeLU [32]. We describe the use of this activation function during the network discussion in Section 3.3.

#### 2.2.2. Fully Connected Neural Network (FCNN)

Layers of nodes in a fully linked neural network are completely connected to one another. A function from $R^{m}$ to $R^{n}$ is a fully connected layer. The fully linked network experiences the vanishing gradient problem since all nodes are interconnected and there may be several more layers [30]. Another problem is the vast array of parameters that fully connecting all nodes of subsequent layers entails. CNNs, on the other hand, are composed of loosely linked neurons rather than fully connected nodes, similar to conventional neural networks. The neurons perform a dot product after receiving some inputs, and then a non-linearity. One goal for these models is to achieve translation invariance, but this is not easily possible with fully connected models. Instead, translation invariance is produced as a result of the repetitive application of several filters in convolutional neural networks [33]. We will discuss these and their limitations below.

#### 2.2.3. Convolutions and Their Limitations

The age-old principle of statistically modeling the link between input features and outputs was used in traditional machine learning. This was exceedingly challenging and did not scale well, as was previously indicated. The introduction of fully linked deep neural networks allowed for the automatic feature extraction from massive datasets. However, CNNs have two significant drawbacks. First, image-based datasets were considered for developing CNNs. The benefit of this is that learnt features like edges, lines, curves, and high- and low-contrast regions can be viewed and portrayed. The learnt properties, however, are incredibly challenging to see or even interpret for datasets of a different kind, like malware.

The second, more significant constraint has more to do with the architecture of CNNs. CNNs are rotation- and translation-invariant by definition. Accordingly, a trait that is found in one place is just as valuable as if it were located in any other place. While this increases the scalability and computational viability of CNNs, it also has some disadvantages. Consider a scenario where an image shows a number of tyres scattered throughout a workshop. The picture also includes a few doors and a car hood. According to CNN, the image satisfies the criteria for what constitutes a “vehicle” because it contains all of those characteristics.

Transformer-based models and later, vision transformers, have been proposed recently to combat this issue. In the following section, we describe the core of vision transformer models, which form the core engine of our technique.

### 2.3. Vision Transformers

Transformers [34] have become the leading architecture for attention-based modeling across various machine learning domains, including both generative and discriminative tasks [9]. They are the foundation for several highly regarded models such as BERT [35], GPT-3 [36], DALL-E [37] and ChatGPT [38], which combine transformer models with reinforcement learning. Extending this concept, vision transformers [7] adapt transformer mechanisms for image modeling. While a detailed mathematical exploration of vision transformers is beyond the scope of this paper, we will provide an overview of the key principles relevant to our work to assist the reader in comprehending our contributions.

All transformer models are based on the concept of the calculation of attention vectors as their core engine. Attention vectors essentially calculate which keys (or features) in the input correspond to the correct prediction of the output. For instance, it is not sufficient that a neural network model predict the image of a car to have the label Car. It is also very instructive that the model outputs exactly which pixels of the input image led to that conclusion. This secondary output is called the attention vector [39]. These attention vectors are computed through the use of gradient descent applied over several types of inputs. These inputs primarily include the following:

- Model input encoded as one-hot vectors in case of text-based inputs and in the form of image patches in case of image-based inputs.
- Position encodings [40] extracted from the inputs. Readers familiar with recurrent models such as recurrent neural networks and Gated Recurrent Unit models would have an understanding of how each input element is fed one-at-a-time to these models. This preserves the spatial relationships between different elements of the input. Transformers, on the other hand, take the whole input as a single vector. Spatial and temporal relations are therefore lost in the process. These relationships are reintroduced through another vector called the position encoding vector, which pre-calculates these relations and is incorporated in the attention mechanisms. Position vectors, thus combined with the input vectors, preserve the Markov property in transformers.

Further, the internal mechanism of transformers includes the following:

- Computation of a second order skip matrix [41] that allows the transformer to keep track of the relationships between, for instance, words in a sentence that are separated by a relative clause. Consider the sentence, The participants, who were interviewed before the experiment began, showed more interest in their questionnaires. In this sentence, the pronoun their is a stand-in for the participants, but is quite far away from it. Second-order sequence models with skips enable the capturing of this relationship.
- Masking of the skip matrices to accentuate the significance of important features attended to by the model. Due to the large number of possible features, even the attention vectors that are computed are severely diluted and are often overwhelmed by the combined weight of large number of insignificant features. Masking solves this issue by effectively gating skip matrix elements that fall below a particular threshold. Without masking, the attention models simply would not work!

**Figure 1:**
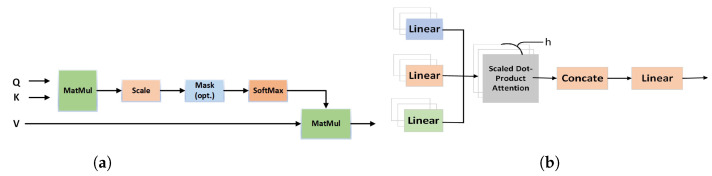
Transformer architecture for multi-head attention model [34]. (**a**) Computation of attention and (**b**) multi-head attention model.

Combining all of these together, as with all deep learning models, is carried out through highly efficient matrix multiplication operations (cf. Figure 1a). In essence, attention is computed through the following formula:

$$\mathrm{Attention}\left(Q,K,V\right)=\mathrm{softmax}\left(\frac{QK^{T}}{\sqrt{d_{k}}}\right)V$$

where *Q* is the query, i.e., the feature of interest and *K* is the collection of masks described above. All possible keys that can be queried are denoted as $d_{k}$ and nthe division by the square root of this value effectively computes a hard maximum, which is then fed to the softmax function to compute probabilities. All possible values that can be held by different keys $K_{i}$ are collected in *V*. This forms the attention model, which is replicated to form the multi-head attention model depicted in Figure 1b. For a detailed discussion about the attention model, we refer the reader to the seminal work on transformers by Vaswani et al. [34]. This multi-head attention mechanism makes the model not only efficient but also explainable, i.e., it is clear why the model is performing the classification instead of it being just a blackbox which gives no insights.

In the following section, we describe how we have modified this exemplary model that has shown exceptional performance images to build an efficient and explainable attention-based model for malware on the Android platform.

## 3. ViTDroid: Proposed Architecture

In this section, we provide the details about our proposed architecture and the rationale for different decisions made during our research. These include the network architecture, embedding choices and different hyperparameters with the aim to enable the interested reader to reproduce our efforts and results on their own.

### 3.1. Network Architecture

Our proposed model—dubbed VitDroid—is designed to utilize the strengths of vision transformers to perform malware analysis for the Android platform. One of the most important goals of this research is to come up with a model that not only performs well in terms of accuracy but also produces results that are explainable. This means that the model should not only output the label—malicious or benign—for an executable target but it must also pinpoint in the opcode sequence the location which contributed significantly to the final result. This explainability would mean that an expert performing the analysis can look into the details of the model output and gain useful insights from the results. This explainability is a major novelty of our work and has not been achieved prior to our work in the domain of Android malware analysis.

**Figure 2:**
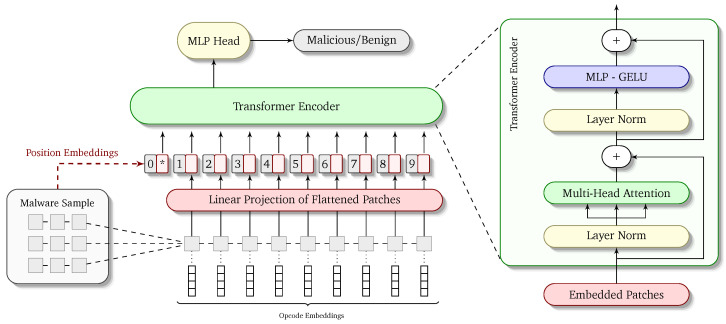
ViTDroid proposed architecture.

In order to achieve this goal, we have created the network architecture in the most modular way possible. The overall architecture of the network is depicted visually in Figure 2. There are two important aspects needed to fully understand the working of ViTDroid: (1) the input to the model in terms of embedded and positionally encoded opcode sequences and (2) the modifications required in the vision transformer model to accommodate our inputs. We describe both of these in the corresponding sections below.

### 3.2. Embeddings for OpCodes

As explained earlier, the standard practice for static malware analysis is to utilize the opcode sequences of executables (both malicious and benign) for feeding into a deep learning model. Since we have previously worked extensively with malware analysis, we have already created a model for the conversion of opcode sequences to low-dimensional vector embeddings. We use the same embeddings as our previous work so that we can make a fair comparison between ViTDroid and previous efforts of malware detection. We note, however, that these previous efforts kept the number of dimensions in opcode embeddings to a minimum to account for the slow training and prediction times of their models.

ViTDroid, on the other hand, due to the use of vision transformers at its core, works in a much more efficient manner and may benefit from a higher-dimensional representation of opcode embeddings. This can help capture finer semantics of opcodes. This forms part of our future work in this area of research. Note also that vision transformers work differently from recurrent neural networks in that there is no back propagation through time (BPTT) to accommodate for the staggered input of sequences elements. The model takes the whole sequence as a single input and the positional information is lost in the process. This information has to be reintroduced through position encodings which we discuss below.

### 3.3. Vision Transformer for Opcodes

Once the inputs are prepared, they are fed to the vision transformer model. Vision transformer is better than plain transformers because it can capture long-range malicious behavior. For example, malicious pieces can be spatially separated in code. We create a 2D plain of 1D opcodes with an empirically selected width of length Ω and varied this hyperparameter to observe the behavior detection changes. Shorter widths can find patters when the spatial separation between two fragments of malicious code is small. On the other hand, with large spatial separation, we need a large width.

We analyzed the behavior of various malicious samples and discovered that optimal analysis requires different values of Ω for different families of malicious applications. This variation is likely due to the distinct latent behaviors exhibited by different malware families. While a more thorough examination of this phenomenon is beyond the scope of this paper, it presents a promising avenue for future research. Our preliminary findings regarding these variations are reported in Section 4.3.

After converting 1D opcode sequences to linear projections of flattened 2D patches, we append positional encodings to the patches. This helps preserve the spatiotemporal relationships between the different opcodes in the input sequence. These positions of encoded embeddings of patches are then fed into the transformer encoder, which forms the heart of the architecture. The embeddings are first normalized using a layer norm. We utilize layer norm instead of batch normalization due to the nature of the input. The usual batch normalization causes a strict adherence to a limited input distribution, whereas our inputs might be from significantly different (and sometimes multi-modal) distributions. We have empirically found layer norm to work more effectively in such cases. After normalization, the embedded patches are fed to the multi-head attention module, which performs the actual learning of attention. We have experimented with several variations of number and sizes of multi-heads. Details about our experiments can been seen in Section 4.3.

After the multi-head attention layers, we add yet another normalization layer in the form of the second level of layer norm and then perform a 2-layer MLP with the Gaussian Error Linear Unit (GeLU) activation function. GeLU is given as

$$\begin{array}{cc} \mathrm{GELU}(x) & =xP(X\leq x)=x\Phi (x)\\ & \;\;=x\cdot \frac{1}{2}\left[1+\mathrm{erf}(\frac{x}{\sqrt{2}})\right] \end{array}$$

where erf is the Gaussian error function given as

$$\mathrm{erf}\left(x\right)=\frac{2}{\sqrt{\pi }}\int_{0}^{x}\exp\left(-t^{2}\right)dt$$

**Figure 3:**
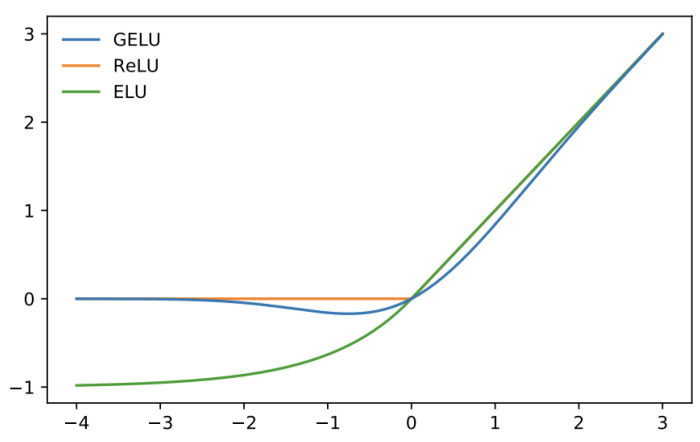
Visual representation of the GELU activation function.

Please refer to Figure 3 for a visual depiction of this activation function. GeLU speeds up the training time as well as the accuracy, owing to the fact that it is smooth and fully differentiable at origin as opposed to ReLU or Leaky ReLU.

#### 3.3.1. Skip Connections

**Figure 4:**
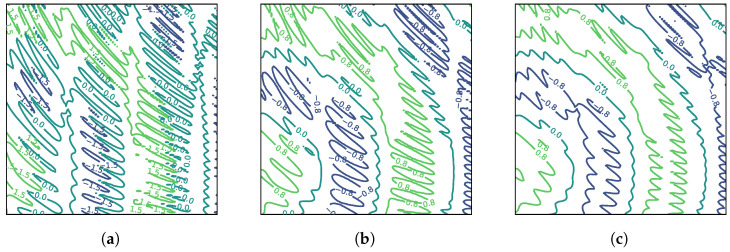
Effect of skip connections on energy landscape of loss in a transformer encoder. (**a**) No skip connection, (**b**) skip connection: k=3, and (**c**) skip connection: k=7.

As is well established, vision transformers are notoriously difficult to train and often lead to very slow iterations. We have solved this issue using skip connections after the multi-head attention layers as well as after the GelU activation. This enabled us to significantly speed up our training while still keeping the accuracy pretty high. Skip connections make the final loss smoother, thus reducing the possibility of becoming stuck in local minima. They also make training much faster by reducing the complexity of the energy landscape. Figure 4 shows how different values of the hyperparameter *k* for skip connections enables us to simplify the energy landscape of our overall loss function, leading to much faster convergence. Notice that these skip connections are different from the skip matrices of the multi-head attention model introduced earlier.

The output of the transformer encoder is then fed to a single-layer perceptron head, which performs binary classification of our inputs into malicious or benign classes. The label is produced as the output of this final layer and the attention matrices are retrieved from the multi-head attention layers from within the transformer encoder. We describe the accuracy of our model’s predictions as well as the efficacy of the attention vectors in Section 4.3. To further speed up the training times and accuracy of our model, we have fine-tuned the vision transformer using some of the most significant state-of-the-art techniques in this domain. In the sections below, we highlight some of these to enable the interested reader to accurately reproduce our results.

#### 3.3.2. Sharpened Cosine Similarity for Feature Extraction

Multi-head attention mechanisms are slow to train due to their nature. Several factors contribute to this lack of speed, so we attacked the problems from different fronts. One of these is to use Sharpened Cosine Similarity [42] during the latent feature extraction phase of the multi-head attention mechanism. This change reduces the number of parameters required for training. This compact variant of vision transformers is inspired from the excellent work presented by Hassani et al. [42].

Sharpened Cosine Similarity is given as

$$scs\left(s,k\right)=\left(\frac{s\cdot k}{\left(\|s\|+q)(\|k\|+q\right)}\right)$$

The parameter *q* is denoting the magnitude of the noise floor expected during training. By adding this parameter to the baseline cosine similarity, we exclude noise from the signal when comparing two signals.

All these features combined enables us to train our model efficiently and produce highly explainable results. We describe the setup of our experiments with this architecture and some of the outcomes from these experiments in the section below.

## 4. Experiments and Results

In order to demonstrate the efficacy of our model and provide a proof-of-concept, we ran several experiments on real-world malware datasets. In this section, we first describe these datasets to enable the reader to gauge the results in a better way. Afterwards, we will describe the performance of our model and the result of changing several control variables.

### 4.1. Dataset Description

Since vision transformers are deep models that are data hungry, we had to find datasets that were both heterogeneous in nature and large in size. We have utilized several publicly available datasets which were combined and then split through random sampling into training and test sets. These datasets include Drebin, containing 5000 malicious applications; the Android Malware Dataset with more than 24,000 samples; and VirusShare, which has more than 10,000 malicious executables. Benign executables were collected from Google Play and checked through VirusTotal [43] to ensure their benign nature.

Combined, we have more than 40,000 malware samples and approximately 50,000 benign applications, thus making the dataset fairly well balanced.

### 4.2. Dataset Preparation

Most datasets available for Android malware (including those used in this study) provide Android package files (APKs) with some metadata. We disassembled these APKs and extracted opcode sequences for each application through AndroGuard (and some tools created in-house). We then used our previously trained embedding model to convert the opcode sequence to their embeddings (cf. Section 3.2).

To keep the inputs as simple as possible, we only utilize the opcode part of each instruction. Operands are discarded as they lead to explosive growth in the computational requirements of neural nets both in opcode embedding creation as well as in the training and prediction pipeline. Moreover, we clipped the opcodes to a maximum of 800,000 (with padding applied to any samples with shorter sequences). This covered around 92% of our executables but enabled us to significantly reduce the training times for our model. The opcode embedding creation was performed one time and the resulting sequences of opcode embeddings were saved in Apache Arrow [44] files. The Apache Arrow standard allows us to use in-memory processing of large-scale samples with minimal code overhead.

We then performed our experiments using the model described earlier. One very important parameter was the width (Ω) parameter of our 2D plane. We experimented with different values of this important hyperparameter. In order to explore the domain space of this hyperparameter paired with the max sequence length parameter, we used grid search. Changing this parameter had significant impact on the results in the obfuscation dataset. We discuss these details in the Section 4.3 below. In the following section, we describe the best results achieved through our experiments as well as some important insights gained from alternative value combinations of different parameters.

### 4.3. Results and Discussion

In this section, we discuss two aspects of our results: (1) a comparative analysis of quantitative results achieved by our model, including accuracy, F1 score and false positive rate; and (2) the explainability of our model and the findings from that front as this forms the major novelty of our work.

**Figure 5:**
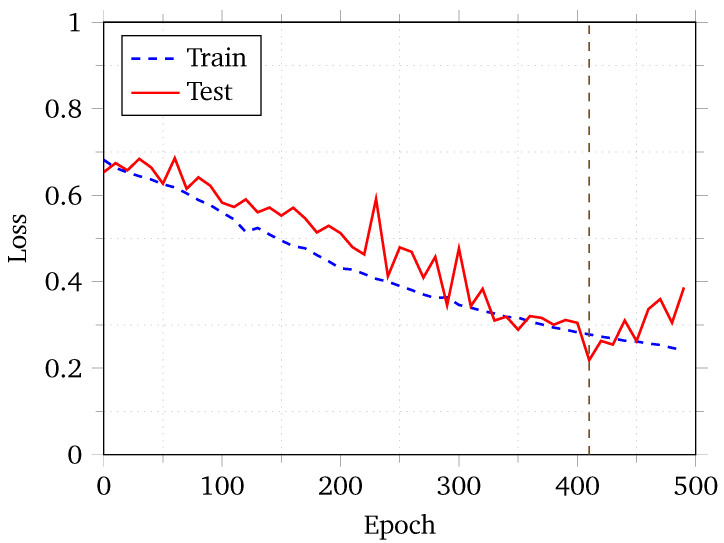
Loss plot for ViTDroid with early stopping.

First and foremost, our model’s loss plot is shown in Figure 5. We ran our model for more than 500 epochs, which grew very steady, and then presented a slow decrease in loss for the first 100 epochs. Afterwards, the neural net seemed to have found a minima and the training loss decreased rapidly until the 400th epoch. After that, while the training loss decreased, the test loss started to increase. Our early stopping mechanism stopped the training at approximately 500 epochs. We used a model checkpoint created at the 410th epoch to calculate the rest of the metrics discussed below.

**Figure 6:**
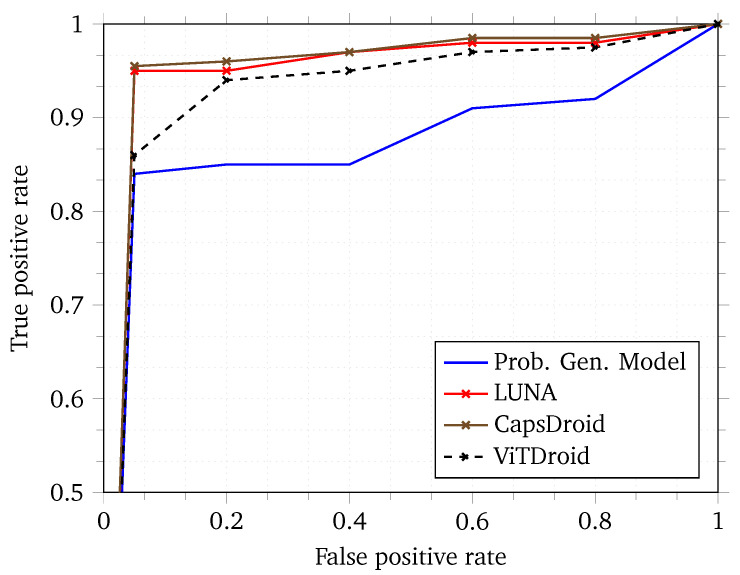
Receiver Operating Characteristic (ROC) curve for ViTDroid and previous models.

To capture the overall efficacy of our model in terms of false positive and false negative rates, we provide a Receiver Operating Characteristic (ROC) curve in Figure 6. The ROC curve captures the behavior of the model based on different thresholds applied to the final softmax layer of the neural network. The higher the Area Under the Curve (AUC) of an ROC, the better the model is in general [45]. As can be seen, ViTDroid performs quite well as compared with previous works. We note that while the false positive rate is high in most cases for ViTDroid when compared to some of the previous works, the delta is quite small. Moreover, the focus of our model is not only on a high AUC but also on explainability. None of the other models achieve this goal of explainability. We believe that the tradeoff between explainability and the AUC is an acceptable one since this can lead to an improvement in future models through further research insights gained through the explainability aspect of our model.

We compare several quantitative metrics of our model’s achieved results in Table 1. Our model’s results are comparable to the state-of-the-art results achieved by [13,14,46], LUNA [26] and CapsDroid [5]. We note that our model does not perform as well as some of the previous works. While the false positive rate (FPR) is lower (i.e., better) for ViTDroid, the F1 score, accuracy and precision have suffered a bit. However, note that the loss is quite acceptable owing to the fact that the model comes with built-in explainability of its results through the attention mechanism. This is a feature simply not present in the other models. As such, this explainability is not only a goal onto itself but may also lead to better and targeted analysis in the future. We believe the tradeoff is well worth the loss in quantitative measures. Moreover, the FPR of our model is slightly better than previous works and this is one measure which is of utmost importance in malware analysis. Future research in this direction seems quite promising to improve the other metrics as well.

**Figure 7:**
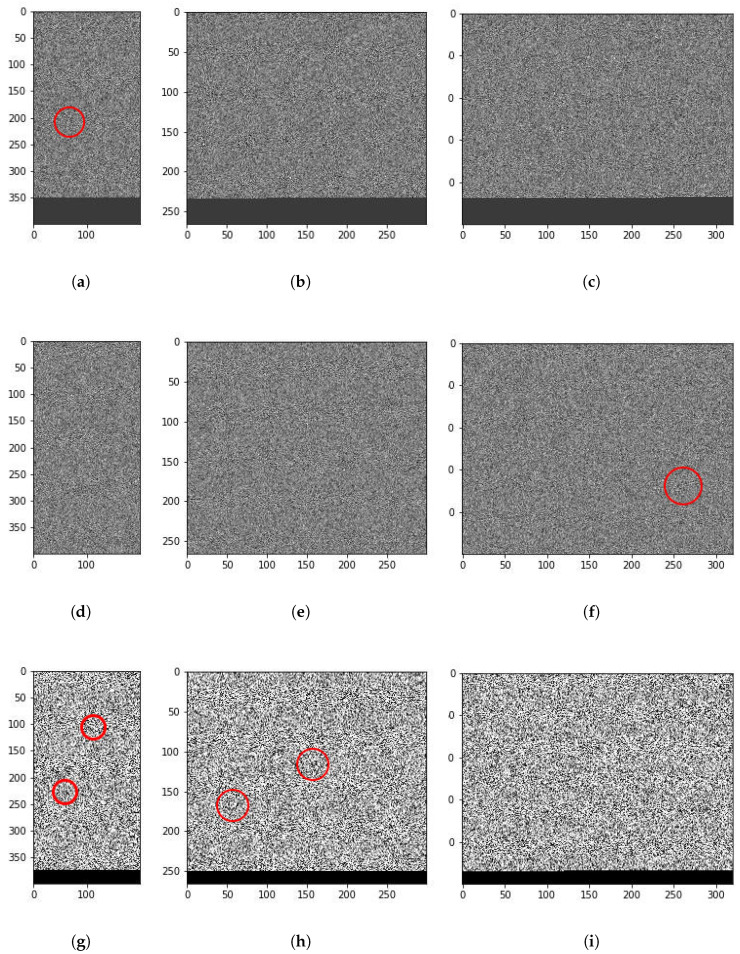
Visual representation of three malware samples—S1: SendPay, S2: FakeRun and S3: SMSReg—at different values of Ω. (**a**) S1: Ω=200, (**b**) S1: Ω=300, (**c**) S1: Ω=320, (**d**) S2: Ω=200, (**e**) S2: Ω=300, (**f**) S3: Ω=320, (**g**) S3: Ω=200, (**h**) S3: Ω=300, (**i**) S3: Ω=320.

Finally, let us discuss the explainability results achieved by our model through the multi-head attention matrices. Figure 7 shows three particular samples of highly common malware families, one in each row. The first one belongs to the SendPay (which is a trojan capable of performing DDoS attacks among other things), the second to FakeRun (adware) and the third to SMSReg (a file infector). We converted their 1D opcode sequence to 2D representation by setting the hyperparameter Ω, which denotes the width of the 2D matrix. Depending on the value of Ω, different interesting features can be identified. For example, there is a thread-like feature in the first sample (Figure 7a). For ease of discussion, we term this feature a filament. A similar but shorter and wider filament can be seen for the third sample in Figure 7g. Another interesting feature is a circular shape visible in Figure 7h. We term such features holes. Both filaments and holes become visible for different values of Ω for certain malware samples. These are only the most obvious features, but there might be several others which might be extracted for different values of Ω. We focus on filaments and holes in particular because our manual analysis of such areas in different malware samples suggests that these do indeed correspond with malicious behavior in code. For instance, we found out through manual analysis that the filament on the left in Figure 7h corresponds to the malicious code segment in SMSReg that reads the device manufacturer ID.

### 4.4. Exploration of Hyperparameters (Ω)

The hyperparameter Ω represents the width of the 2D plane onto which the 1D opcode sequences are projected. Different values of Ω are crucial for effectively capturing the spatial structure of opcode sequences for various malware families. To investigate the optimal value of Ω, we conducted a grid search over a range of values (e.g., 100, 200, 300, 400) across different malware datasets.

Our experiments revealed that different malware families benefit from varying Ω values due to their unique behavior. For instance, malware with highly compact malicious code performed better with smaller Ω values (e.g., 200), allowing the model to focus on closely related opcodes. In contrast, malware with disjointed or long-range dependencies required larger Ω values (e.g., 320), which helped the model capture patterns to spread across larger portions of the code.

Table 2 summarizes the performance (in terms of accuracy, F1 score and false positive rate) for different Ω values across various malware families.

These results show that Ω values influence the model’s ability to capture malicious patterns effectively. A higher value of Ω works well for malware families with dispersed malicious code, while smaller values are sufficient for more localized behaviors.

**Figure 8:**
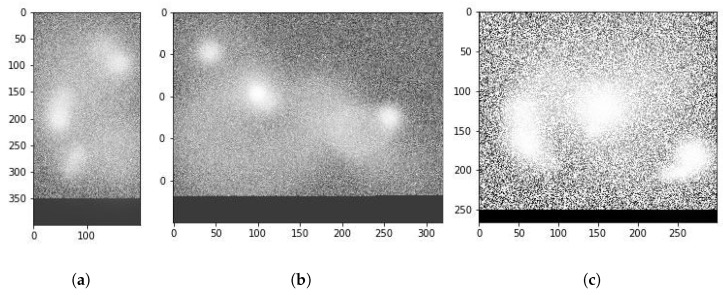
Example attention maps learned for different combinations of malware samples and Ω variations. (**a**) S1: Ω=200, (**b**) S1: Ω=320, (**c**) S3: Ω=300.

A change in Ω also results in different areas being identified by the model. We observed in the extended results generated by our model that different values of Ω work for different malware families. Families which have malicious behavior extended over long periods seem to work best with larger Ω values. More obviously malicious malware is detected well with lower Ω values as well. This aspect of our work requires an extensive study in its own right and forms part of our future plans of exploration. Note that actual attention matrices are quite complicated. To aid in the explainability of our model, we first trained it using a multi-head attention model with 18 heads. After the training was completed, we retrieved the attention matrices of only the most prominent heads to plot. For instance, Figure 8 shows a visual representation of this attention matrix for the first sample shown in Figure 7. As can be seen, the attention model has indeed identified the interesting malware behavior that was known a priori by us. Moreover, it has also identified some other interesting areas that can be a target of expert analysis for further study.

Such visual analysis make sense of the complicated attention maps, as can be seen, in Figure 8. The mapping results of all heads may simultaneously point out even more points of interest, but the visualization cannot be easily visually perused by humans. Note that the model also tends to pay attention to those areas in the binary image map, where there are either filaments or holes present. The presence of these structures in a particular area seems to be a necessary but not a sufficient condition for the attention head to focus on the area.

### 4.5. Explainability Through Attention Mechanisms

One of the key advantages of using vision transformers in malware detection is the explainability they provide through attention maps. Unlike CNNs, which function as blackbox models, ViTs allow us to visualize which parts of the input data (in this case, opcode sequences) contributed most to the classification decision.

In Figure 4, we provide examples of attention maps generated by our model for both benign and malicious Android binaries. As shown, the model highlights specific opcode sequences in red, indicating their contribution to the classification as malware. For example, in a SendPay trojan (Figure 4a), the attention map focuses on sequences related to unauthorized access and data transmission.

These attention maps allow security experts to trace back the model’s decision and understand which parts of the code are most likely to be malicious. This not only improves detection accuracy but also assists in manual code analysis by highlighting high-risk areas that require further investigation.

### 4.6. Practical Insights from Attention Maps

The attention maps generated by our model offer valuable insights into the structure and behavior of malware. In practical terms, these maps can help malware analysts identify suspicious behavior patterns more quickly and accurately. For instance, in the SMSReg family (Figure 7g), the model’s attention map highlights a region of the code responsible for reading sensitive device information—a known malicious behavior.

Further, the adjustability of attention-related parameters such as the number of heads or the Ω value enables our model to focus on different areas of interest, depending on the malware family. By adjusting these parameters, analysts can fine-tune the model’s focus on either broad behavioral patterns or specific malicious instructions, depending on the analysis required. The practical implication of these insights is significant. Attention maps reduce the time and effort required for the manual inspection of code by directing experts to the most relevant parts. This contributes to faster detection and deeper understanding of newly emerging malware.

### 4.7. Formal Methods for AI-Based Technique Verification

In addition to standard performance metrics, formal methods can provide important guarantees about the correctness and safety of AI-based techniques like ViTDroid. Formal verification methods, such as model checking, can be applied to validate that the model adheres to certain safety properties or constraints, ensuring that its decisions are reliable and trustworthy. Incorporating formal methods into AI-based malware detection would allow security practitioners to verify that the model’s predictions meet rigorous standards for accuracy and safety. Future work will explore the integration of formal verification techniques to further enhance ViTDroid’s trustworthiness in critical applications.

## 5. Conclusions and Future Work

Malware analysis on the Android platform is not only complicated but also a highly challenging and moving target due to the nature of the platform and the heterogeneous ecosystem. It is not sufficient to be able to classify newly emerging malware. It is also important for human experts to understand the underlying nature of newly emerging malware. For this reason, the latest trend of throwing data at deep learning models and accepting the produced results is actually counter-productive despite its recent successes. In this paper, we have presented a novel model dubbed ViTDoid that is not only able to classify malware with high accuracy but also produces results that are explainable. The attention mechanism built into our model provides focused results which help the malware analysis expert find out which portion of the full executable is actually the cause of the malicious behavior. We understand that this is a new venue of research and a lot of study has to be carried out to further the understanding of how this model can be used to identify behaviors of different malware groups and even to identify new families. All of this forms part of future work along this line of research.

## Figures and Tables

**Table 1:** Comparison of results with existing techniques.

Metric	Prob. Gen. Model [13]	Drebin [46]	Obf. Resilient [14]	LUNA [26]	CapsDroid [5]	ViTDroid
Accuracy	-	-	0.858	0.989	0.991	0.951
Precision	-	-	0.892	0.982	0.983	0.984
F1 Score	-	-	0.874	0.986	0.987	0.976
Coverage	1.0	1.0	1.0	0.976	1	1
FPR	-	0.01	-	0.0021	0.0021	0.0019
Detec. Rate	-	0.94	0.969	0.981	0.980	0.982
AUC	0.953	-	-	0.983	0.984	0.976

**Table 2:** Performance metrics for different Ω values across various malware families.

Ω Value	Accuracy	F1 Score	False Positive Rate
200	0.951	0.976	0.0021
300	0.965	0.983	0.0019
320	0.971	0.984	0.0018

## Data Availability

Malware data used in this study are publicly available from their respective owners. References have been provided throughout the manuscript to enable the reader to reach out to their original authors.
